# 2327 Decoding/encoding somatosensation from the hand area of the human primary somatosensory (S1) cortex for a closed-loop motor/sensory brain-machine interface (BMI)

**DOI:** 10.1017/cts.2018.60

**Published:** 2018-11-21

**Authors:** Brian Lee, Richard Andersen, Helena Chui, William Mack

**Affiliations:** University of Southern California

## Abstract

OBJECTIVES/SPECIFIC AIMS: A brain-machine interface (BMI) is a device implanted into the brain of a paralyzed or injured patient to control an external assistive device, such as a cursor on a computer screen, a motorized wheelchair, or a robotic limb. We hypothesize we can utilize electrical stimulation of subdural electrocorticography (ECoG) electrodes as a method of generating the percepts of somatosensation such as vibration, temperature, or proprioception. METHODS/STUDY POPULATION: There will be 10 subjects, who are informed, willing, and consented epilepsy patients undergoing initial surgery for placement of subdural ECoG electrodes in the brain for seizure monitoring. ECoG will be used as a platform for recording high-resolution local field potentials during real-touch behavioral tasks. In addition, ECoG will also be used to electrically stimulate the human cerebral cortex in order to map and understand how varying stimulation parameters produce percepts of sensation. RESULTS/ANTICIPATED RESULTS: To determine how tactile and proprioceptive signals are integrated in S1, we will perform spectral analysis of the broadband local field potentials to look for increased power in specific frequency bands in the ECoG recordings while touching or moving the hand. To explore generating artificial sensation, the subject will be asked to perform a variety of tasks with and without the aid of stimulation. We anticipate the subject’s performance will be enhanced with the addition of artificial sensation. DISCUSSION/SIGNIFICANCE OF IMPACT: Many patients might benefit from a BMI, such as those with stroke, amputation, spinal cord injury, or brain trauma. The current generation of BMI devices are guided by visual feedback alone. However, without somatosensory feedback, even the most basic limb movements are difficult to perform in a fluid and natural manner. The results from this project will be crucial to developing a closed loop motor/sensory BMI.

